# Uncharted territory of the epidemiological burden of cutaneous leishmaniasis in sub-Saharan Africa—A systematic review

**DOI:** 10.1371/journal.pntd.0006914

**Published:** 2018-10-25

**Authors:** Temmy Sunyoto, Kristien Verdonck, Sayda el Safi, Julien Potet, Albert Picado, Marleen Boelaert

**Affiliations:** 1 Department of Public Health, Institute of Tropical Medicine, Antwerp, Belgium; 2 Policy Department, Médecins Sans Frontières - Campaign for Access to Medicines, Geneva, Switzerland; 3 Faculty of Medicine, University of Khartoum, Khartoum, Sudan; 4 ISGlobal, Barcelona Institute for Global Health, Barcelona, Spain; Institut Pasteur de Tunis, TUNISIA

## Abstract

**Introduction:**

Cutaneous leishmaniasis (CL) is the most frequent form of leishmaniasis, with 0.7 to 1.2 million cases per year globally. However, the burden of CL is poorly documented in some regions. We carried out this review to synthesize knowledge on the epidemiological burden of CL in sub-Saharan Africa.

**Methods:**

We systematically searched PubMed, CABI Global health, Africa Index Medicus databases for publications on CL and its burden. There were no restrictions on language/publication date. Case series with less than ten patients, species identification studies, reviews, non-human, and non-CL focused studies were excluded. Findings were extracted and described. The review was conducted following PRISMA guidelines; the protocol was registered in PROSPERO (42016036272).

**Results:**

From 289 identified records, 54 met eligibility criteria and were included in the synthesis. CL was reported from 13 of the 48 sub-Saharan African countries (3 eastern, nine western and one from southern Africa). More than half of the records (30/54; 56%) were from western Africa, notably Senegal, Burkina Faso and Mali. All studies were observational: 29 were descriptive case series (total 13,257 cases), and 24 followed a cross-sectional design. The majority (78%) of the studies were carried out before the year 2000. Forty-two studies mentioned the parasite species, but was either assumed or attributed on the historical account. Regional differences in clinical manifestations were reported. We found high variability across methodologies, leading to difficulties to compare or combine data. The prevalence in hospital settings among suspected cases ranged between 0.1 and 14.2%. At the community level, CL prevalence varied widely between studies. Outbreaks of thousands of cases occurred in Ethiopia, Ghana, and Sudan. Polymorphism of CL in HIV-infected people is a concern. Key information gaps in CL burden here include population-based CL prevalence/incidence, risk factors, and its socio-economic burden.

**Conclusion:**

The evidence on CL epidemiology in sub-Saharan Africa is scanty. The CL frequency and severity are poorly identified. There is a need for population-based studies to define the CL burden better. Endemic countries should consider research and action to improve burden estimation and essential control measures including diagnosis and treatment capacity.

## Author summary

Cutaneous leishmaniasis (CL) is the most common form of this group of parasitic diseases, transmitted by sandflies. In sub-Saharan Africa, its extent of the problem is unknown, while elsewhere its disfigurement and stigma may cause a severe impact. This study systematically searched the literature to find evidence on the epidemiological data on human CL in this part of the world. Historically, CL has been present for decades in both western and eastern Africa, but unfortunately, in the last decades, the data are irregular and patchy. The estimated burden, relying on detected cases, may only capture part of the true number of cases. This article shows that there is insufficient evidence to have accurate figures; the diversity of the disease, along with poor surveillance have resulted in unprecedented CL outbreaks in the past. Many knowledge gaps remain, and we highlight the importance of improving the current fragmented knowledge by increasing commitments to tackle CL and conduct better population studies. CL in sub-Saharan Africa appears to be a blind spot and should not remain so.

## Introduction

Cutaneous leishmaniasis (CL) is the most common clinical manifestation of leishmaniasis, a parasitic neglected tropical disease (NTD) [1]. Caused by an obligate intracellular protozoa from the *Leishmania* species and transmitted by the bite of Phlebotomine sand flies, the clinical presentations of CL include localized skin nodules (often called oriental sores), diffuse non-ulcerated papules, dry or wet ulcers, and, in the mucocutaneous form, extensive mucosal destruction of nose, mouth, and throat. Transmission of CL may involve animal reservoir hosts (e.g., rodents, hyraxes) in zoonotic foci, while anthroponotic CL (where humans are the main parasite reservoir) occurs in urban or periurban settings [2]. Environmental changes in rural contexts such as agricultural activities, irrigation, migration, and urbanization may increase the exposure risk for humans and result in epidemics. Likewise, outbreaks in densely populated cities or settlements have occurred, especially in conflict-affected zones such as Afghanistan or Syria [3,4], in refugee camps and contexts of large-scale forced migration of populations.

Globally, the World Health Organization (WHO) considers CL as endemic in 20 countries in the New World (South and Central America) and in 67 countries in the Old World (southern Europe, Africa, the Middle East, parts of southwest Asia) [5]. Between 700,000 to 1,200,000 CL cases are estimated to occur annually worldwide, with >70% of cases in 2014 reported from Afghanistan, Algeria, Brazil, Colombia, Costa Rica, Ethiopia, the Islamic Republic of Iran, Peru, Sudan and the Syrian Arab Republic [5,6]. Multiple parasite species cause CL: in the Old World, these are *L*. *major*, *L*. *aethiopica*, *L*. *tropica*, and, rarely, the viscerotropic *L*. *donovani* (in Sudan), resembling similar a phenomenon more known for *L*. *infantum* [7–10]. Though CL is often considered self-healing, the duration varies for different species and can take months, or years [11].

Due to the clinical and epidemiological diversity in CL, its geographic clustering and lack of reliable surveillance data, estimating the CL burden are challenging [12]. The most widely used measure of disease burden known as the Disability Adjusted Life Year (DALY) combines estimated prevalence, incidence, and mortality, with an assigned disability weight for each disease [13]. However, the disability weights are defined using different approaches with regards to the expert panel composition, health state description, and valuation methods [14,15]. The specific stigma and psychosocial distress generated by a non-fatal condition are often overlooked [16,17], although the social impact of CL is potentially severe and has been well-documented [18,19].

Moreover, in sub-Saharan Africa (SSA), not only the disability but also the number of CL cases is largely underestimated. A recent global burden analysis listed 19 countries in SSA in the top 50 high burden countries [20]. The passive epidemiological surveillance system that prevails in these countries leads to the patchy data from this region. According to WHO, only Sudan and Ethiopia reported cases of CL [21]. The objective measures of burden such as prevalence and incidence of CL are scarce in this region, making it hard to advocate for funding and resources to tackle the disease.

Whereas attention has been given to CL in Northern Africa (Algeria, Libya, Morocco, Tunisia, Egypt) and the Middle East [22–24], data for sub-Saharan Africa is critically lacking, particularly in countries where CL is not a notifiable disease. This study focuses on SSA because it is a blind spot on the CL epidemiological burden map and the overall picture of what has been documented on CL is not known. We undertook a systematic review of the literature to synthesize current knowledge on CL burden in SSA.

## Methods

### Search strategy and selection criteria

We searched the following electronic databases: National Library of Medicine through Pubmed, Cochrane Register, Web of Science, CABGlobal Health, African Index Medicus and Google Scholar. We did an initial keyword search and subsequent searches based on Medical Subject Headings (MeSH) with various combinations of search terms “cutaneous leishman*” AND “Africa, South of the Sahara” (which also included “Africa, Western”; “Africa, Eastern”; and “Africa, Southern”) OR “Leishmaniasis, cutaneous” OR “Leishmaniasis, diffuse cutaneous” OR “Leishmaniasis, mucocutaneous” AND each individual sub-Saharan countries. The World Bank classification was used to define sub-Saharan African countries and to group them according to the region (i.e., southern, eastern, western, and middle Africa- see Box 1). No language restrictions were set for searches, while we limited the publication date until 31 May 2018. We hand-searched the reference lists of all recovered studies for additional references. We also explored and summarized information from the Global Health Observatory for leishmaniasis maintained by WHO for CL.

**Box 1. Countries of sub-Saharan Africa**

Angola, Benin, Botswana, Burkina Faso, Burundi, Cabo Verde, Cameroon, Central African Republic, Chad, Comoros, Democratic Republic of Congo, Republic of Congo, Cote d’Ivoire, Equatorial Guinea, Eritrea, Ethiopia, Gabon, (the) Gambia, Ghana, Guinea, Guinea-Bissau, Kenya, Lesotho, Liberia, Madagascar, Malawi, Mali, Mauritania, Mauritius, Mozambique, Namibia, Niger, Nigeria, Rwanda, Sao Tome and Principe, Senegal, Seychelles, Sirra Leone, Somalia, South Africa, South Sudan, Sudan, Swaziland, Tanzania, Togo, Uganda, Zambia, Zimbabwe

We included studies if they are reporting primary data that help to determine the burden of CL in countries in SSA. The burden is defined as elements of 1) severity of the problem (clinical, disability, case fatality,…) in human patients; 2) frequency (prevalence, incidence,…) and 3) economic cost (from patient, societal or health system perspective). We excluded animals or vector studies, studies on pathogenesis, immunology, histopathology, or on *Leishmania* species only, studies on diagnostic tests or treatment for CL and cases of Post Kala Azar Dermal Leishmaniasis (PKDL)–skin sequelae of VL. Case reports and case series of fewer than ten patients were also excluded. Sub-Saharan Africa as the main geographical interest refers to the settings where the studies were performed/conducted. Reviews about CL in a specific country or region without original data were excluded.

The systematic review was conducted in line with PRISMA guidelines [25,26]. The review protocol was registered in PROSPERO, an international prospective register of systematic reviews, in July 2016, number 42016036272 [27].

We selected the articles in a two-step process. In a first stage, titles and abstracts of all retrieved records were independently reviewed by two investigators (TS and KV). In a second stage, the selected full-text articles were again reviewed (by TS, KV, and a third person) for eligibility. When full-text articles were excluded, the reason for exclusion was registered and reported. Any discordances were resolved through discussion or seeking consensus with a third investigator (MB).

## Data extraction and synthesis

The data were extracted in parallel by two independent readers, using a specific data form, including information on the published record (year, author), setting (country), aim, study design, and main outcomes. We sought data on prevalence or incidence of CL among patients in health facilities and the community; demographic and clinical characteristics of CL patients, and the association between CL and other morbidities, notably Human Immunodeficiency Virus (HIV). We attempted to use the STROBE checklist (for reporting epidemiological studies) to assess the ‘risk of bias,’ but could not continue due to a large number of historical studies that are not in line with current reporting standards. The data analysis thus resulted in a narrative, qualitative synthesis of the included studies.

## Results

### Search results

**Fig 1:**
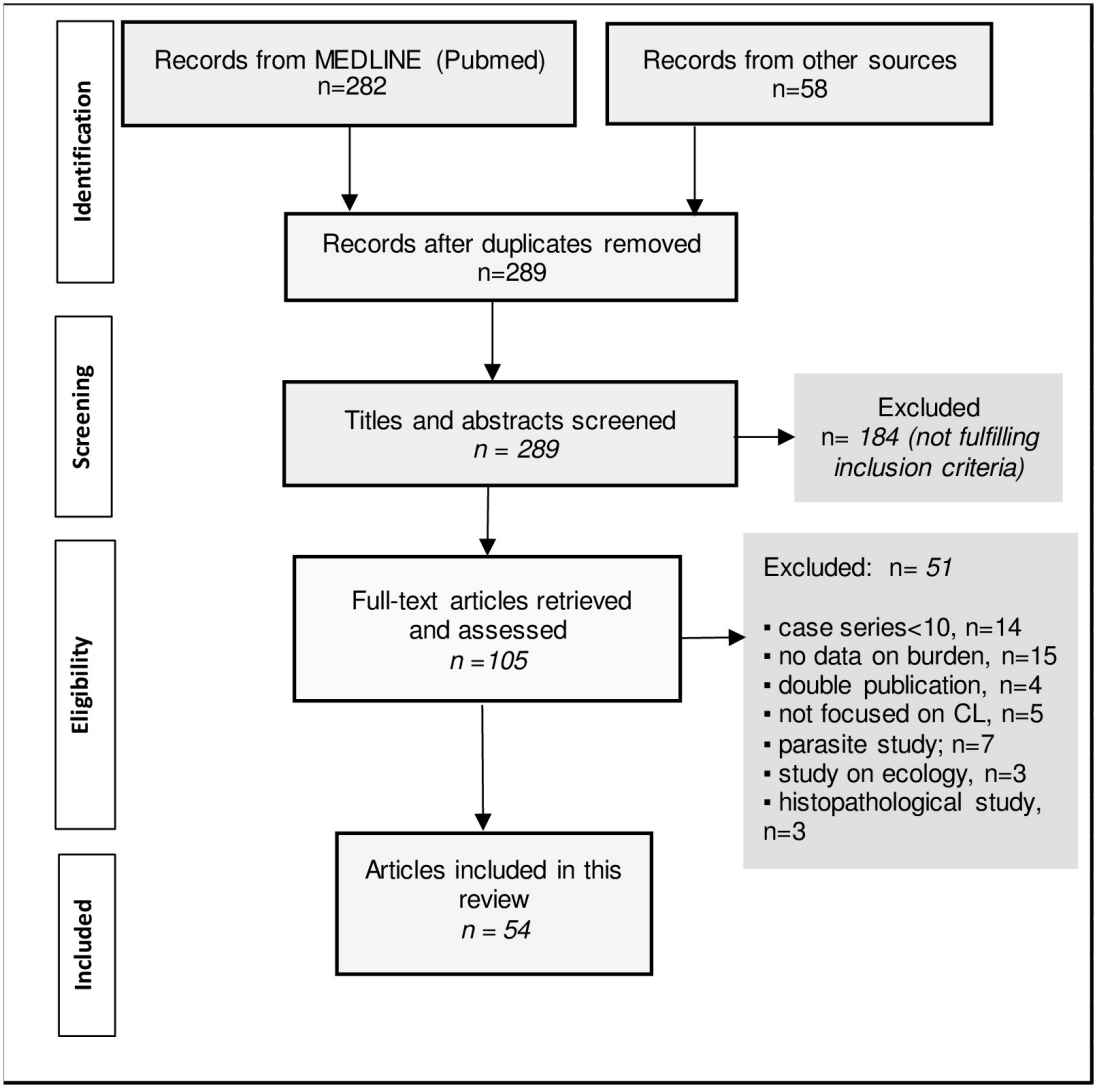
Flow diagram depicting the selection of eligible articles.

The flow diagram in Fig 1 shows the selection process: we identified 340 published articles, and after removing duplicates, we screened the title and abstracts of 289 articles, and exclude 184. The full-text articles of the remaining 105 were assessed for eligibility, after which a further 51 were excluded. The remaining 54 articles were included. (See *Supporting Information 1* for all the included studies and the key information).

### Description of the included studies

The studies were published between 1955 and 2016; with only 12 (22%) after 2010. The studies were conducted in 13 out of the 48 countries in Sub-Saharan Africa: in eastern Africa (Ethiopia, Kenya, Sudan), western Africa (Burkina Faso, Cameroon, Chad, Ghana, Guinea, Niger, Nigeria, Mali, Senegal) and southern Africa (pre-independent Namibia). More than half of the studies were from western Africa (30/54), notably Senegal (6), Burkina Faso (5) and Mali (5). Twenty-three studies studied CL in the community (including three among school-children), and 28 used data collected in health facilities (including 18 dermatology specialized services). The remaining three studies were mixed. All 54 studies were observational: 29 (54%) were descriptive case series (numbering a total of 13,257 cases), and 25 (46%) followed a cross-sectional design, usually survey with various tools employed such as clinical screening or questionnaires.

### Historical accounts of cutaneous leishmaniasis in sub-Saharan Africa

In eastern Africa, CL has been known for more than a century, with the first indigenous CL case recorded in 1911 in Sudan [28]. In Ethiopia, CL has been known since 1913, and diffuse CL (DCL) clinical form was documented in 1960 in the highlands [29]. The first report of *L*. *aethiopica* as a distinct taxonomic entity was published in 1978 [30,31], and since then, the species has also been found in the mountainous region of Kenya [32]. *L*. *tropica* was later reported from certain areas in Kenya during the 1990s, and since then considered to have a more restricted distribution than *L*. *major* [33,34].

In western Africa, only *L*. *major* has been thought to circulate in this region. The oldest case reports of CL come from Niger in 1911 [35], then from Nigeria in 1924, and from Senegal in 1933 [36]. Later more cases were reported from Cameroon, Mali, Mauritania, Burkina Faso and Guinea [37,38]. During the first half of the 20^th^ century, the colonial medical officers documented sporadic case reports from an area that later became recognized as the ‘CL belt’ [38]. Several comprehensive ecological and epidemiological studies took place in suspected hyperendemic foci in Senegal [39–42], Mali and Niger [43]. Current Namibia (previously South West Africa), reported dozens of CL cases in the 1970s [44], but the disease was not considered as a public health problem by the authorities [45].

### Exposure to the parasite: Frequency of leishmanial infection measured through population surveys

Twelve studies (Table 1) reported prevalence estimated by the Leishmanin Skin Test (LST)—also known as Montenegro test—to detect exposure to the parasites in CL foci. Through intradermal injection of *Leishmania* antigens, the induration is being read 48–72 hours later as a demonstration of a delayed type hypersensitivity reaction, much like a tuberculin skin test [11]. LST does not differentiate between past and present infection and not species specific, yet it is often used as a marker for cellular immunity against CL [46].

**Table 1:** Overview of studies describing the frequency of exposure to *Leishmania* based on Leishmanin Skin Test (LST).

Region	Author, Year, [Ref]	Study year	Country, Location	Setting	Number of people subjected to LST	The proportion of positive LST results
**Eastern Africa**	Mengistu, 1992 [49]	1989	Ethiopia, Ocholo (west Rift Valley)	Community	120	57%
	Berhe, 1998 [51]	1994–1996	Ethiopia, mid-Ethiopian Rift Valley	Community	1809	3%
	Kadaro, 1993 [48]	1990	Sudan, Khartoum province	Community	1479	91%
	Abdalla, 1973 [47]	NA	Sudan (Blue Nile, Kartoum, Darfur)	Community	560	22%
	Abdalla, 1975 [52]	NA	Sudan, eastern part	Hospital	15 (cases)	80%
**Western Africa**	Pampiglione, 1977 [37]	1976	Guinea, Kamsar	Community	388	15%
	Imperato, 1970 [43]	1969	Mali, Nioro in Kayes region (western)	Community (school)	550	61%
	Imperato, 1974 [53]	1973	Mali,Mopti (central)	Community (school)	249	5%
	Oliveira, 2009 [50]	2006–2008	Mali, Segou district (central)	Community	1530	31%
	Traore, 2016 [54]	2014	Mali, central/western and southern	Community	1412	39%
	Dedet, 1979 [55]	1976–1978	Senegal, Thies Region	Community	NA	58%
	Dedet, 1979 [56]	1978	Senegal, Fleuve Region	Community	1489	47%

These studies were conducted at the community level in CL foci, and have shown fluctuation over time (Table 1). Changes from 4% to 91% in LST positivity rate were observed in the same villages following an outbreak in Sudan [47,48]. High variability across foci within one country has also been reported, for example in Ethiopia: in Ocholo, 57% of school children without CL lesions were LST positive [49], while another study in the central-Ethiopian Rift Valley, LST positivity was maximum 5%. A study conducted in two neighboring villages in central Mali also demonstrated high variability: prevalence of *Leishmania* infection in Kemena was 45%, with the incidence of 19% and 17%; higher than Sougoula with 20%, 6% and 6% for the same years [50]. Reasons for these discrepancies are not known but possibly linked with hyper-clustering of reservoirs and vectors, leading to different intensity of peridomestic transmissions in Kemena [50].

A 2014 study from Mali complemented LST surveys with PCR and finger prick blood sample to measure antibody levels to sand fly saliva in endemic districts [54]. The results showed uneven prevalence of LST positivity across three different climatic areas (49.9%, 24.9% and 2.6% in Diema, Kolokani, and Kolondieba respectively), linked with north-south declining vector density. PCR was used to confirm *L*. *major* as the causative agent. LST positivity was also shown to be correlated to higher levels of antibodies to sand fly salivary proteins [54].

Across the studies, a consistent finding is that the proportion of positive LST increased with age and areas where CL transmission is active, at least a third of the population have had exposure to the *Leishmania* parasite [37,43,47–51,54–56].

### Prevalence and incidence of cutaneous leishmaniasis in sub-Saharan Africa

Twenty-one studies reported estimates of CL prevalence or incidence; five were using medical records from hospitals, and the remaining were population estimates obtained through active screening for CL lesions and scars at the community level. All diagnosis was based on clinical examination. Though additional confirmatory methods (microscopy/smear, histology, culture in NNN or combination of these) were mentioned in all studies but two, it is unclear whether these were used in some or all or none of the patients. Among the five studies that were hospital-based, two used the number of dermatology consultations as the denominator, and the CL cases proportion found is 2% in Ouagadougou, Burkina Faso [57] and 14% in Addis, Ethiopia [58]. If suspected cases were to be denominator to calculate the CL cases proportion, they were found to be 78% (251/320) in Mali [59] and 93%(74/80) in Burkina Faso [60].

In most of the studies in the community, the prevalence of active CL was less than 5%. In endemic areas, the frequency of CL scars usually exceeds that of CL active lesions, except in a few special settings (Table 2). In Utut, Rift Valley in Kenya, a higher lesion versus scar rate (50% vs. 18%) in migrant charcoal workers suggested a non-immune population’s encounter with the disease in an area where transmission occurs [34]. Also during an outbreak in a new focus in Silti, Ethiopia, the frequency of CL lesions was considerably more than that of CL scars [63]. In Sudan, 36% of the community were found to harbor active lesions during an outbreak [68].

**Table 2:** Prevalence and incidence of active lesions and scars of cutaneous leishmaniasis.

Region	Author, Publication Year, [Ref]	Country, Location	Setting	Number of people screened	Prevalence CL (active lesion)	Prevalence CL scars	Incidence
**Eastern Africa**	Wilkins, 1972 [61]	Ethiopia, Meta Abo	Community	1635	0.6%	3.2%	0.1%
	Lemma, 1969 [62]	Ethiopia, highlands	Community	>2000	2.9%	2.9%	
	Negera, 2008 [63]	Ethiopia, Silti (SNNPR)	Community	1907	4.8%^A^	0.3%	
	Mengistu, 1987 [64]	Ethiopia, Ocholo (southwest)	Community	2689	6.0%	40.0%	
	Mengistu, 1992 [49]	Ethiopia, Ocholo	Community	3022	3.8%	34.3%	
	Bsrat, 2015 [65]	Ethiopia, eastern Tigray	Community	2106	7.1%	6.9%	
	Bekele, 2014 [58]	Ethiopia, Addis Ababa	Hospital	1651	14.2%		3.5%
	Sang, 1993 [66]	Kenya, Mt Elgon	Community	1979^A^	1,3%		
	Sang, 1993 [67]	Kenya,Nairobi+Rift Valley	Community	3743	0.5%	0.3%	
	Sang, 1994 [34]	Kenya, Utut	Community	167	49.7%	18.0%	
	Abdalla, 1978 [68]	Sudan, Shendi Atbara	Community	308	36%^B^		
			Dispensaries	NA	20–50%		
	Kadaro, 1993 [48]	Sudan, Khartoum province	Community	458	4.0%	47.0%	
**Western Africa**	Bamba, 2013 [57]	Burkina Faso, Ouagadougou	Hospital	12708	2.0% ^C^		
	Guiguemdé, 2003 [60]	Burkina Faso, Oudagougou	Hospital	80	92.5% ^D^		
	Keita, 2003 [59]	Mali, Bamako	Hospital	320	78.0%^E^		0.6%
	Obasi, 1991 [69]	Nigeria, Kaduna	Hospital	18000	0.1%^F^		
	Ngouateu, 2012 [70]	Cameroon, Mokolo (north)	Community	32466	0.4%	0.8%	
	Oliveira, 2009 [50]	Mali, Segou district (central)	Community	1530			9.4%
	Okwori, 2001 [71]	Nigeria, Kaduna	Community	10226	3.9%	3.0%	
	Ikeh, 1994 [72]	Nigeria, Keana	Community	5046	3.9%		
	Dedet, 1979 [73]	Senegal, Thies region	Community	1049	3.7%	8.7%	0.2%

NA- Not Available;

^A^ General survey outside the survey’s two villages yield prevalence of 0.1% (18/18528);

^B^ This study was done during an outbreak (see text)

^C^ During 1999–2007; 251 confirmed CL cases among all consultations in the Dermatology Service of University Hospital

^D^ Confirmed CL amongst suspected cases (74/80). Also reports the prevalence of CL and HIV

^E^ During 1997–2001; 251 confirmed CL cases among suspected file

^F^ During 1979–1988; 21 CL cases among 18,000 dermatology consultations in Ahmadu Bello University Teaching Hospital

**Fig 2:**
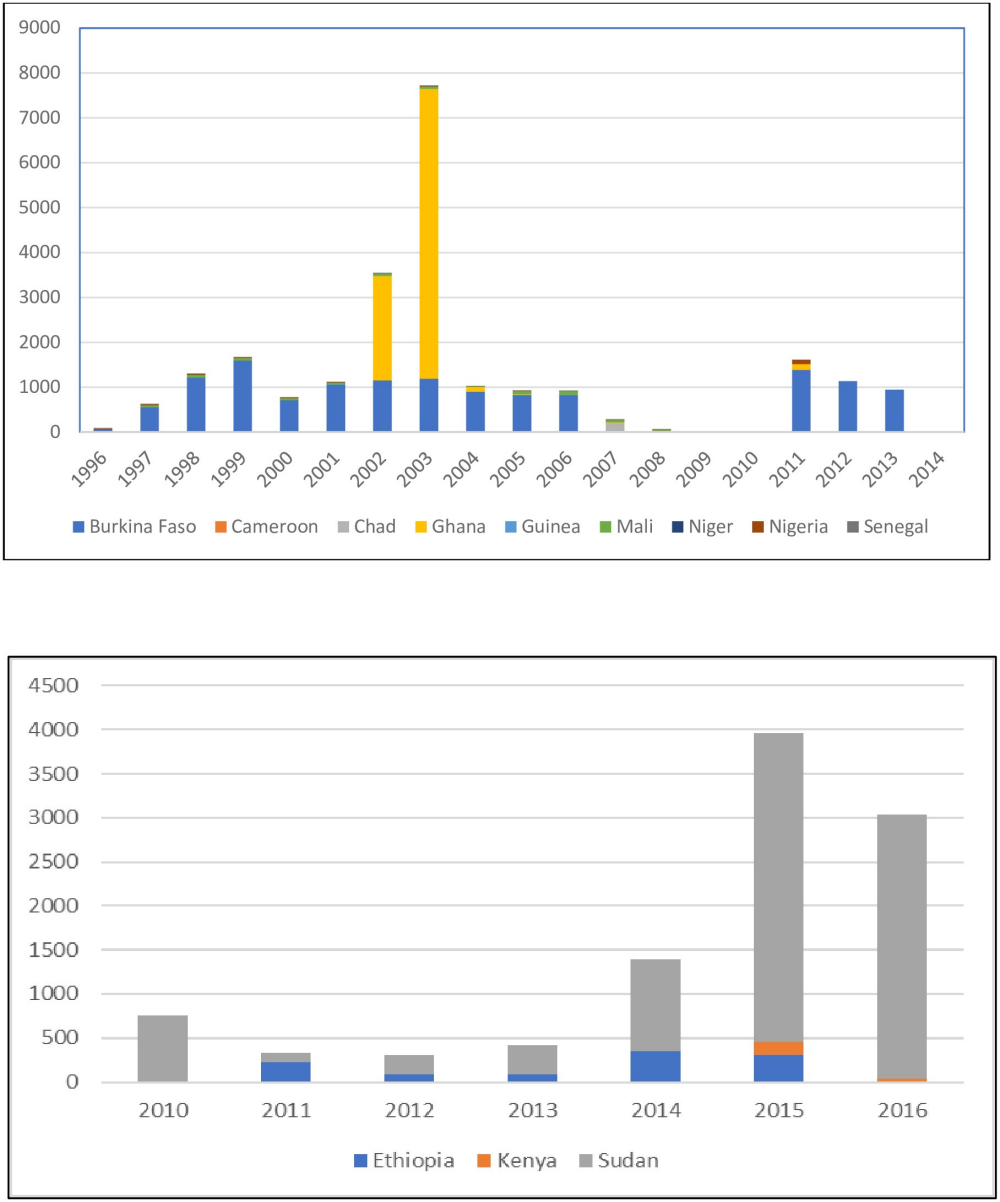
A.) Reported cutaneous leishmaniasis cases in western and central Africa, WHO Global Health Observatory. B.) Reported cutaneous leishmaniasis in eastern Africa, WHO Global Health Observatory.

To complement the findings from published studies, we also examined the data from the country official reporting system to WHO. The system record data from 1996 onwards, but clearly there are missing data (Fig 2A and 2B). The absolute number of CL cases reported from eastern Africa is always higher than from western Africa, with Sudan bearing most of the burden. In western Africa, the number of cases reported from different countries is highly variable, and recurrent outbreaks were occurring in a 5–7 years cycle [74]. The increased cases in Ghana during 2002–2003 was prominent, yet there was a vacuum between 2007 and 2010, and cases were reported again starting in 2011. Other countries contribute little, with <100 cases per year (Nigeria, Senegal). No data was reported from this region during 2015–2017 [75].

### Cutaneous leishmaniasis case series

The majority (n = 28) of the included records are clinical case series based on medical files from dermatology clinics or hospitals as the main data source. These studies describe a cohort of CL patients over a certain period, ranging from two to nine years. Chronologically, 10 studies reported CL cases in periods before 1980 [41,45,47,52,74,76–80], 11 described patient groups observed between 1980–2000 [35,57,59,67,69,81–87], and seven between 2000 and 2013 [58,60,88–92].

Hospitals reported that CL patients mainly came from surrounding areas or outside the cities or capital, such as Dakar, Senegal [74,88,93] or Niamey, Niger [84]. Eighteen studies report cases seen in specialized dermatology services. The proportion of CL cases among patients seen in those dermatology clinics is consistently less than 5% [59,69,94]. In the context of an outbreak, CL patients who seek care in specialized services represent only the tip of an iceberg, as shown in Burkina Faso (further described below). Between 1999 and 2005, a total of 7444 cases were recorded from various health centers in the capital Ouagadougou [95,96], but during the same period, the dermatology hospital had only seen 251 CL cases [57]. Diagnosis in all the case series is obtained through clinical examination and smears or histopathology. In Chad, a hospital close to the Sudanese border reported a very high proportion of CL confirmed cases (580 out of 680 cases between 2008–2012) [89].

### Cutaneous leishmaniasis outbreaks

Three countries have published studies on CL outbreaks: Sudan, Ethiopia, and Ghana. The first ever epidemics in Sudan were reported in 1976–1977 along the Nile, in Shendi-Atbara north of Khartoum [68], while the second and third outbreaks occurred in 1985 and 1986–1987, respectively [97]. The last epidemic in Sudan was in Tuti island, and it affected at least 10,000 people in 7 months. Underestimation is likely mandatory reporting only started after the epidemic reached its peak [86]. People of both sexes, all age groups and all socio-economic classes were affected, which is suggestive of a disease ravaging in a non-immune population. The causal parasite was *L*. *major* LON-1 [98] and the outbreak was attributed to various factors such as immigration from west Sudan, the heavy rainfall in the year of the outbreak after a long period of drought—which led to increase in sandfly density as well as the rodent reservoir population—and waning of herd immunity of migrants from CL endemic areas in western Sudan (*Sayda el-Safi*, *personal communication*). In Ethiopia, a CL outbreak occurred in 2005 in a district 150 km south of Addis. A survey then established an overall prevalence of 4.8% (92/1907), and 1 in 5 cases had mucocutaneous lesions [63].

In Ghana, an outbreak of localized skin lesion consistent with CL occurred in Ho municipality, Volta region in 2003 [90]. The usual triggers of CL epidemics such as intrusion of humans into vector habitat through deforestation, road construction, wars or migration were not at work here. Previously, only one CL case had been reported from the country in 1999, although the arid, Sahelian area of northern Ghana is considered to be part of the West African CL belt. Through passive case detection (with biopsy as a confirmatory diagnosis) with medical records review and active case finding, it was estimated that there were about 8876 CL cases between 2002 and 2003 in Ghana (Fig 2A). All age groups were affected, and since then CL is considered endemic in this area. A study in the same district later found 60% parasite-confirmed cases among active CL suspects (41/68). A phylogenetic analysis identified this Ghanaian parasite as new member of *Leishmania enriettii complex*, a possible new subgenus of pathogenic human Leishmania parasites [99].

### Clinical aspects of cutaneous leishmaniasis

Thirty-two studies described the clinical presentations of CL lesions. The most commonly used categories of the lesions are as followed: the localized CL or LCL, otherwise known as the classic oriental sore, refers to the lesion at the site of sand fly bites that may get ulcerated. LCL may appear as dry, papular forms with crust, or the wet, ulcerative forms with indurated edges. LCL can be singular or multifocal. When the nodules are multiple and nonulcerative, this is typically called a diffuse CL or DCL. In Sudan, mucosal leishmaniasis is described as lesion(s) that involves destructive mucosal inflammation which does not always start with a cutaneous lesion. This differs from New World mucocutaneous leishmaniasis (MCL), which refers to a metastatic dissemination to the mucosal tissues starting from a distal cutaneous lesion [52,100]. Bacterial superinfection is common along with pain, itchiness, fever and the secondary inflammation often complicates clinical diagnosis [11,101].

The diagnosis documented in the medical files are often missing. A dermatology hospital in Addis, Ethiopia reported that among 234 confirmed CL cases, only 22% were categorized—consisting of 9% DCL, 10% MCL and 3% LCL [58]. The higher proportion of complicated or atypical lesions are frequently reported from teaching hospitals or specialized services. This includes sporotrichoid CL with painless subcutaneous nodules along the lymphatic vessels in Sudan [80,87], or the diffuse CL in Ethiopia, which appear pseudo-lepromatous and can result in fungating or tumor-like lesions [52,80].

In the majority of the studies, the natural history of the lesions is only briefly described (n = 51). The duration between the first bite to lesion formation for LCL varied between 3–12 weeks [62,90]. Although CL can heal spontaneously, this seems to be dependent on the reported parasite species: *L*. *major* heals within approximately 2 to 12 months and *L*. *tropica* within 15 months, with a terminal scar appearing after about 24 months [11]. The description of diffuse CL caused by *L*. *aethiopica* suggests that it presents initially with nodules which do not heal or ulcerate but can metastasize widely [76] and are known to be very difficult to treat. In the case of DCL, spontaneous cure almost never happens. Mucocutaneous leishmaniasis is rare in Africa, but cases have been reported from Sudan and Ethiopia [52,80,100]. The lesions tend to be infiltrative and result in chronic edematous inflammation involving the lips, nose, buccal mucosa and larynx are.

With regard to the locations of CL lesions, there appears to be a regional difference. CL lesions from eastern Africa are mostly found on the head (i.e., face including cheek, nose, forehead, ears, lips) and less on the arms, legs or trunk, while from western Africa the highest proportion of lesions are on the upper and lower extremities.

**Fig 3:**
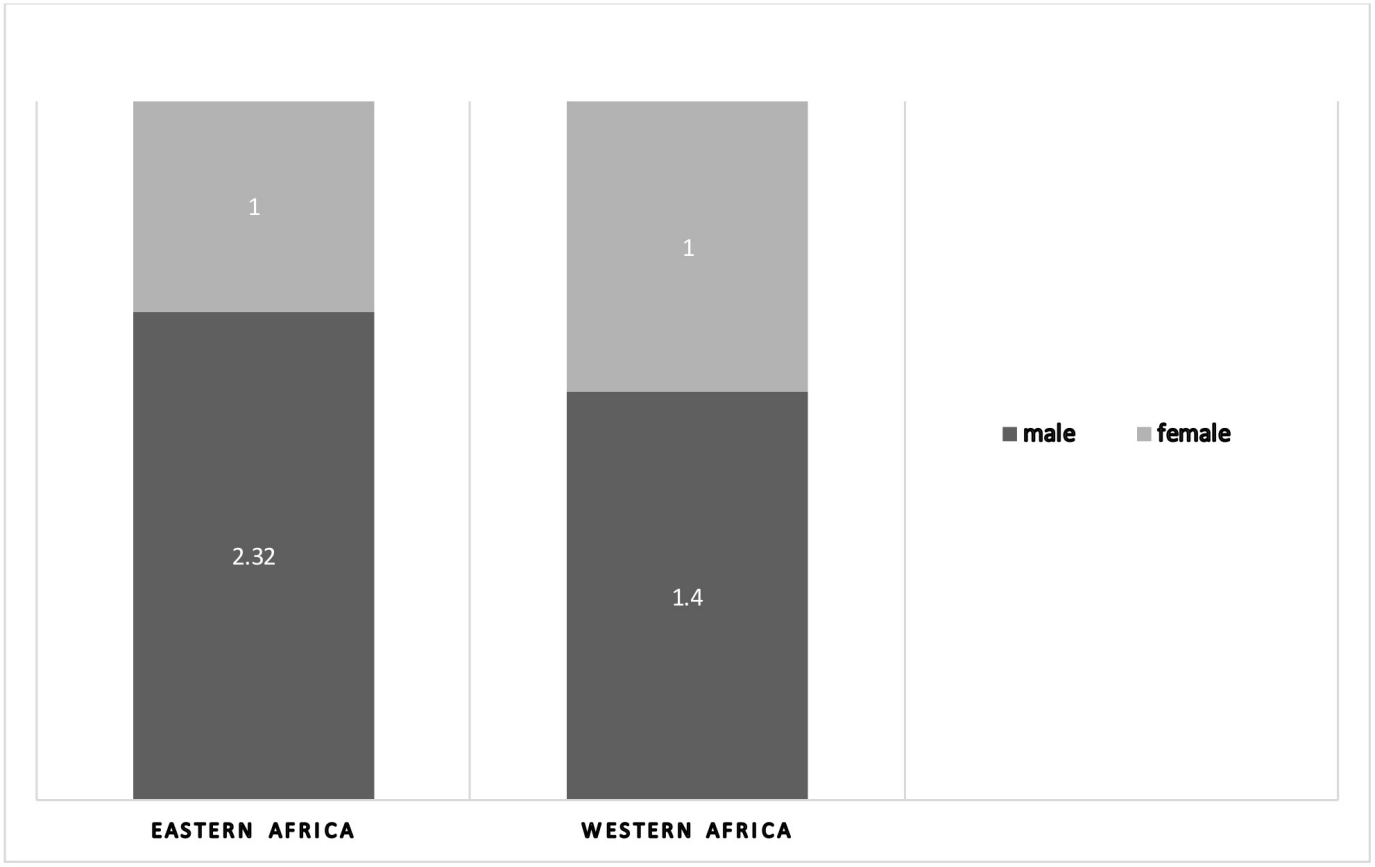
Sex ratio among cutaneous leishmaniasis cases in sub-Saharan Africa. Source: Studies included in this review that reports sex ratio amongst the CL cases (n = 18 studies from eastern Africa; n = 24 studies from western Africa).

Amongst the 42 studies reporting the sex ratio of the patients (Fig 3), only 12 recorded more females than males affected [49,50,56,63,70,72,82,95,102] while the remaining described male preponderance, either due to hypothesized occupational exposure or males’ easier access to seek care in a health facility. Thirty-six out of the 54 studies reported the age of the CL cases: people of all ages are affected. However, when stratification according to age was reported, there is a broad tendency towards younger age groups (between 10–30 years old.

### Cutaneous leishmaniasis co-infection with Human Immunodeficiency Virus (HIV)

CL and HIV co-morbidities has been described in Burkina Faso [57,60,103], Cameroon [70], Mali [59], and Ethiopia [91], while sporadic cases have also been reported from Guinea, Ghana, Senegal, Nigeria, Ivory Coast and Sudan. Burkina Faso has recorded 13.5% (10/74) HIV positivity in a cohort of CL patients in 2000, and another cohort of 32 CL/HIV patients was described in 2003–2004 [60,103]. Six out of 10 DCL cases in Ouagadougou were co-infected with HIV [57]. In Bamako, Mali, the prevalence of HIV among CL patients was 2.4% [59]. In Tigray, Ethiopia, a study reported an HIV prevalence of 5.6%, which increased to 8% two years later in 167 CL patients [92,104]. The only study reporting CL/HIV prevalence in the community was done in Cameroon in 2008. Here, a total of 32 466 subjects were clinically screened, and amongst 146 active CL patients, seven (4.8%) tested positive for HIV-1 and/or HIV-2 [70].

The consistent finding is that the clinical forms of CL are more diverse and complex in HIV co-infected patients, posing significant challenges in diagnosis and treatment. The lesions tend to be more severe: there are reports of infiltrative, leprosy-like, diffuse, psoriasis-like, verrucous, sporotrichoid, and angiomatous or Kaposi-like. Patients are more likely to have more than one lesion and more than one clinical forms [103]. Also, the time to lesion healing was longer in immunosuppressed individuals [70], and particularly in atypical and severe CL patients with poor response to treatment [91].

## Discussion

Our review shows that CL is reported in at least 13 countries in SSA but the true burden remains unknown. Several foci in Mali, Guinea, and Senegal have been studied intensively in the last half of the 20^th^ century, but the published literature on CL can best be described as irregular and patchy. There is a lack of population-based or longitudinal studies to measure prevalence and incidence. The current CL burden is difficult to estimate accurately as primary data are scarce and CL cases often clusters in pocket areas. The prevalence in western Africa appears to be low, yet unprecedented outbreaks have occurred, such as in Burkina Faso and Ghana. Several CL outbreaks probably never get reported [105,106]. In eastern Africa, although the number of CL cases are high, there is insufficient evidence on CL prevalence and incidence outside the context of CL outbreak or its spread to new areas.

The findings from this review provide further insights vis-à-vis the official data reported to the WHO’s global surveillance system. Based on reported cases in 2002–2009, WHO estimated a global CL incidence of 214,036 in 2012 with 35,300–90,500 cases from eastern Africa and a mere 790–1500 cases from the rest of SSA, albeit with 5–10 fold underestimation [5]. Data reported to WHO in 2005–2015 put the figure of global CL incidence at 187,855, and the estimated contribution of SSA remains negligible [107]. From the 2013 Global Burden of Disease (GBD) study which primarily used modeling, Sudan and Burkina Faso are the only two countries from SSA with significantly greater DALYs from CL than the global mean [20]. Our findings are in line with these, thus emphasising the critical need to improve on-the-ground data as sources for future estimates.

The quality of evidence found in our review is inadequate to establish a more accurate CL burden in this region. Case series provide a snapshot of a specific situation in a certain time and place, yet are hard to extrapolate. A considerable part of the data we reviewed originated from specialized dermatology services which only represent a small proportion of all CL cases. The patchwork distribution of CL within a country further hampers surveillance. The CL belt in SSA from West Africa to the Horn of Africa [38], confirmed with a modeled distribution map of CL [108], appears to be mainly supported by historical accounts. The currently available evidence is clearly limited.

Various factors have been attributed to the poor CL data from SSA [2,12,109]: 1) CL is not a notifiable disease in many of the endemic countries; 2) Patients do not seek care due to perceived self-healing nature of CL; 3) Poor access to health facilities as most affected people live in remote, rural areas; 4) Lack of control tools, including unavailability of diagnosis and limited capacity to offer effective treatment. Compared to other regions, the neglect of CL is obvious. For New World CL in Latin America, the Pan American Health Organization (PAHO) has coordinated efforts to standardize and centralize surveillance data [110]. A Regional Information System called SisLeish was eventually developed to become an essential tool to prioritize areas and guide control actions [111]. Understandably, the region bears a much higher burden than SSA (from 2001–2015, 843931 cases were reported from 17 countries in the Americas). Currently there is no regional approach to improve CL surveillance for SSA. Sudan is part of the WHO Eastern Mediterranean Region (EMRO) [112] while the rest of the SSA countries belong to the WHO African Region (AFRO).

Our review identified the fragmented knowledge on burden as one of the key challenges for CL control in SSA. Being a largely zoonotic disease, the control efforts for CL remains limited to care provision, while vector control or environmental measures are not feasible. The risk of outbreaks, however, should not be undermined. Co-infection with HIV, already a concern for VL, might pose further challenges in CL management. What can be done in the face of all these adversities?

In lights of the scanty data, steps should be taken to improve existing surveillance systems or establish one where it is non-existent. Each country could undertake a thorough review of CL epidemiological situation, using standardized methods, enabling compilation and comparison. The future actions must be adjusted to the country context. An integrated paradigm should be adopted: either in setting up rapid epidemiological assessments for CL alone or in taking opportunities to include CL with other skin-NTDs [113,114]. Recognising the common challenges of a vertical approach to each NTD affecting the skin, a common tool to monitor disability has been piloted [115]. Furthermore, WHO has recently released guidelines for the training of skin NTD for frontline health workers [116,117]. Building capacity in case detection through training or inclusion of CL in clinical guidelines is starting in Sudan and Ethiopia, following an algorithm developed for Eastern Mediterranean region by WHO [118].

The strengths of this review are the systematic search of the literature and the stringent process and reporting following a published protocol in PROSPERO. Furthermore, standardized reporting according to PRISMA guidelines is adhered to. The exclusion criteria for case series of fewer than ten patients have been chosen as the aim is to provide an idea on disease burden though we might risk missing individual case report(s) and may exclude countries which only has case report publications. By systematically assessing all published articles we aimed to draw attention to the importance of the disease and identify research priorities.

The major limitations of our study are first, the publication bias. Sub-national studies that are not published nor listed in the international electronic databases might be missed. Secondly, the weakness of passive detection and clinical case reporting. We could not provide a meta-analysis nor compare the results between studies, due to the high variability across individual studies (denominator, sampling strategy, …). We could not systematically assess the risk of bias in the individual records and apply the current standard of as many studies pre-dated this era. The quality of the data in the studies is relatively poor. However, with the limited data we had to rely on, we understand better the state of the evidence in regards to CL in SSA: still an uncharted territory.

Based on the gaps identified in this review, there are some research priorities to be addressed (see Table 3). Improving epidemiological knowledge on CL will help to advocate for actions and resources in SSA, where the burden of NTDs surpass all other regions [119]. Future studies on CL burden should explore not only physical but also the socio-economic impact of this morbidity. CL in sub-Saharan Africa should not remain an enigma.

**Table 3:** Major topics on CL epidemiology and burden in sub-Saharan Africa identified in this review.

Research topic	Total number of identified studies	Comment
CL incidence	5	Better field data and regular, standardised reporting
Outbreak-associated with CL	3	Outbreaks are often overlooked and not documented
Risk factors for CL	0	Important to inform health messages and design control
The social impact of CL	0	The psychosocial distress has never been reported here
Economic burden of CL	0	Access barriers and access to care need to be prioritized
Factors that sustain transmission of CL	0	More studies needed on transmission dynamics of CL (vector, reservoir, hosts)

### Conclusion

The epidemiological burden of cutaneous leishmaniasis in sub-Saharan Africa appears to be poorly documented. There is a paucity of robust evidence on prevalence and incidence on CL in this region. The diversity of CL epidemiological characteristics in endemic countries is not yet fully investigated. Nevertheless, the burden of CL morbidity remains important and most likely to be underestimated. Surveillance and mapping should be improved to mitigate outbreak risk and address dual co-infection with HIV. The current fragmented knowledge should be approached regionally, and awareness must be raised. In addition to population-based studies that better define the CL burden in sub-Saharan Africa, health systems should consider studies and action to improve CL essential diagnosis and care.

## Supporting information

S1 Diagram PRISMA flow diagram. (DOC)

S1 Checklist PRISMA checklist. (DOC)

S1 Table Key information from the studies included in this review. (DOCX)
