# Supplementary material for: Uncharted territory of the epidemiological burden of cutaneous leishmaniasis in sub-Saharan Africa—A systematic review
Source: PLoS Negl Trop Dis. 2018 Oct 25;12(10):e0006914. doi: 10.1371/journal.pntd.0006914 (PMC6219817; doi:10.1371/journal.pntd.0006914)
Supplement: S1 Diagram — (DOC) [file pntd.0006914.s001.doc]

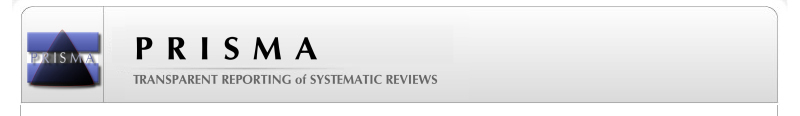
**PRISMA Flow Diagram**

**Screening**

**Included**

**Eligibility**

**Identification**

Records identified through database searching
(n = 282 )

Additional records identified through other sources
(n = 58 )

Records after duplicates removed
(n = 289 )

Records screened
(n = 289 )

Records excluded
(n = 184 )

Full-text articles assessed for eligibility
(n = 105 )

Full-text articles excluded, with reasons
(n = 51 )

Reasons:

▪ case series<10, n=14

▪ no data on burden, n=15

▪ double publication, n=4

▪ not focused on CL, n=5

▪ parasite study; n=7

▪ study on ecology, n=3

▪ histopathological study, n=3

Studies included in qualitative synthesis
(n = )
