# Supplementary material for: Uncharted territory of the epidemiological burden of cutaneous leishmaniasis in sub-Saharan Africa—A systematic review
Source: PLoS Negl Trop Dis. 2018 Oct 25;12(10):e0006914. doi: 10.1371/journal.pntd.0006914 (PMC6219817; doi:10.1371/journal.pntd.0006914)
Supplement: S1 Table — (DOCX) [file pntd.0006914.s003.docx]

| Study | Country | Study design | Study setting | Characteristic(s) of study participants | Number of cases reported | Prevalence/Incidence reported | Diagnostic method | Parasite(s) | Clinical presentation | Male/Female ratio |
| --- | --- | --- | --- | --- | --- | --- | --- | --- | --- | --- |
| Abdalla et al, 1973 | Sudan | Case series | Civil Hospital | Patients | 21 | 0.22% LST+ | Smear + histopathology | NA | nodular/noduloulcerative, ulceratuve, diffuse | 9.5:1 |
| Abdalla et al, 1975 | Sudan | Case series | Hospital |  | 51 | LST+ 12/15 | Smear + blood + histopathology | NA | oral, nasal, oro-nasal, laryngeal | Only male |
| Abdalla et al, 1978 | Sudan | Cross-sectional | Community | along the Nile | 308 | 29% skin lesions in Trajama, 59% skin lesions in Kijeik | Smear | NA | multiple, nodulonulcerative/nodular, mean è-28 lesions | NA |
| Bamba et al, 2013 | Burkina Faso | Case series | dermatology service of CHU YO | Dermatology patients | 251 | 251CL/12708 patients. 6VIH/10DCL | Smear | L major assumed | crusted40%; papuloulcerated16% | 0.89:1 |
| Bamba et al, 2011 | Burkina Faso | Case series | Health centers of city Ouagadougou | Patients | 7444 (1999-2005) | 0.1%+-0.04* | NA | NA |  | 0.89:1 |
| Bekele et al, 2014 | Ethiopia | Case series | Dermatology service | Dermatology patients. 53% rural, 47% urban. | 234 | 234 cases/1651 suspected cases | Microscopic examination | NA | plaque, ulcer with nodules | 1.3:1 |
| Berhe et al, 1998 | Ethiopia | Cross-sectional | Community |  |  | 36positive/1167reported | LST |  |  | NA |
| Bryceson et al, 1969 | Ethiopia | Case series | Leprosarium | Patients | 33 | NA | Smear, NNN | NA | single lesion not ulcerate, spread during 4m to 11 y (mean 3 y), not involving mucosa except 4. Extensive graphics. Involvement of lymphoedema and lymphadenopathy. Comorbidities such as filariasis and leprosy | 1.75:1 (among whose are sick, but it was not proportional) |
| Bsrat et al, 2015 | Ethiopia | Cross-sectional | Community |  | NA | P. 14% | Clinical examination + smear and culture NNN | L aethiopica |  | NA |
| Dedet et al, 1982 | Senegal | Cross-sectional | Community |  | NA | Inc. 1976 : 3.31/1000, 1977 : 0.98/1000, 1978 : 0.26/1000. Prev 1978 : 12.39% scars 8.67%, active case 3.71%. Immunity : 57.8% | Physical examination + LST | NA |  | 0.68:1 |
| Dedet et al, 1982 | Senegal | Case series | Clinic | Patients | 60 (1976 : 24, 1977 : 30, 1978 : 6) |  | NNN | NA | no halo de Faye, ulcerated with thick crusts, 11% protruding,. 25% has lymphatic trajectories | 1.4:1 (* Men are more likely to go to town). Urban : 1.2:1 |
| Dedet et al, 1979 | Senegal | Cross-sectional | Community |  | 1489 | 695/1489 LST+ | LST | NA | NA | 1.5:1 |
| Develoux et al, 1990 | Niger | Case series | Dermatology clinic | Dermatology patients | 64 |  | Smear + Biopsy | L major assumed | dry ulcerocrusted and humid ulcerous, multiple, 10 with lymphangitis | 2:1 |
| Develoux et al, 1991 | Niger | Case series | Dermatology center of Boukoki | Dermatology patients | 64 confirmed (/96 suspected) |  | smear + Histology | NA | ulcer crust, pseudosporotritrichosic, pseudotumoral | 2:1 |
| Diop et al, 2016 | Senegal | Case series | Dermatology department of the Social Hygiene Institute | Dermatology patients | 87 recensed, 50 included | 3 diabetes /50CL | Clinical + parasitology + histology | NA | ulcerative crusty, spporotrichoid, ulcerative w secondary infection | 1.6:1 |
| Djibrilla-Kaou et al,1979 | Cameroon | Case series + cross sectional | Community + "Centre de Médecine Préventive" |  | 58 |  | Microscopical examination + smear | NA | nodule which ulcerates in the middle, always evolving and leaving scars | 1.15:1 |
| El-Safi et al, 1991 | Sudan | Case series | Hospital for Tropical Diseases | Patients | 736 | 23/736 have diabete, 18% have a secondary infection | Smear | L major LON-1 | multiple. ulcerative44%, nodulouulcerative31%, nodular31%. 11%lymphatic. Itching61%, pain38%fever17%, secondary inf18% | 1.32:1 |
| El Safi et al, 1991 | Sudan | Case series | Commission of Health Affairs | Non-immune population? | 736 |  | Smear + Culture | L major LON-1 | typical of L major | 1.56:1 |
| Gaafar et al, 1994 | Sudan | Case series | Dermatology clinics + community | Dermatology patients | 177 SUD + 100 S.AR. |  | Biopsy + clinical examination | L major | multiple noduls, painless, occasionally ulcerated | 1.08:1 (SUD), 5.67:1 (S.AR.) |
| Grove et al, 1989 | South Africa |  |  |  | 34 |  | Skin biopsy | L major MON-74 |  | NA |
| Grove et al, 1978 | South Africa | Case series |  | Patients | 18 | NA | Histologic | NA | painless red papules to large ulcers | NA |
| Guiguemde et al, 2003 | Burkina Faso | Case series | Dermatological units | Dermatology patients | 80 | 74 confirmed cases/80 patients ; 10 co-infected with HIV/80 | Biopsy + NNN + Smear | NA | Chronic non healing ulcers | 1:1 (LC); 2.33:1 (Co-infection) |
| Ikeh et al, 1994 | Nigeria | Cross-sectional | Community | Farmers | 5046 examined | 4.5% female lesions, 3.2% mle lesions. 197/5046 active lesions | Clinical examination + microscopy | NA | papular, fungating, ulcerating-multiple, painful and itchy, healed ulcers leave tissue-paper thin scars | 0.71:1 |
| Imperato et al, 1974 | Mali | Cross-sectional | Ecole Fontamentale | Students |  | 12/249 positive reactions | LST | NA | NA | 2.5:1 (Positive rate) |
| Imperato et al, 1970 | Mali | Cross-sectional | Niori's School | Students | 550 | 61.3% | LST | NA | NA | 1.88:1 |
| Jeliffe RS, 1955 | Nigeria | Case series | Outpatient deparment City Hospital + ?? | In and Out-patients | 23 |  | NA | L tropica assumed | sores | NA |
| Kadaro et al, 1993 | Sudan | Cross-sectional | Community | Volunteers (?? Bad traduction??) | Population : 1479 | 12/303 had active lesions, 19/303 had no active lesions + negative rate, 276/303 had a positivity rate + 147/303 had scars | Smear + LST | NA |  | 1:1 |
| Keita et al, 2003 | Mali | Case series | Dermatology-lepro service of CNAM | Dermatology patients | 251 | I:6.27/1000. HIV:6/251diabete: 1/251 | Physical examination + smear/biopsy | NA | multiple in 200, single in 50 | 3:1 |
| Kodindo et al, 2015 | Chad | Case series | Hospital | Patients |  | 580positive/680 exam | Laboratory | NA | NA | 1.7:1 |
| Kone et al, 2012 | Mali | Cross-sectional | Community | 49/50 Dogon (+60% unemployed) | 50 suspected cases |  | Blood, smear, biopsy | L major MON25 26 | multiple ulceration, w or w o crust | 1.38:1 |
| Kweku et al, 2011 | Ghana | Case series, survey, seeking | Community + Volta Regional Hospital | School-children farmer, trader, students, teacher, self-employed |  | <2003 : 2426, 2003 : 6450 cases | Smears + biopsy | L major MON 26 117 74 | pustule, nodule ulcerated | Female dominant (case series) |
| Lariviere M, 1966 | Senegal | Case series | Dermatology service, clinic | Dermatology patients | 39 |  |  | NA | polymorphous (oriental sore; ecthymatous, ulcer, dry/psoriasis like/lichenoid, lupoid | 1.29:1 |
| Lemma et al, 1969 | Ethiopia | Cross-sectional | Community | Peasant farmes, school-children, villagers | NA | 57/2000 with active lesion & 58/2000 with scars. No new cases of DCL | Intradermal inoculation | NA | nodular, later ulcerate, scars very characteristics | Male dominant |
| Mengistu et al, 1992 |  | Cross-sectional + Propesctive | Community | Ocholo Farmer's Association |  | P active LCL in 1987 : 3.55%, in 1989 : 3.97%. Positivité in 68% active lesions, 61% scars | Smear + NNN | NA | slight bleeding and local pain- fungating tumor in oral mucosa, or diffuse ulcerative lesions in nasal mucosa | 01:01 |
| Mengistu et al, 1987 | Ethiopia | Cross-sectional | Community | Ocholo Farmer's Association |  | 53CL/146Elephantiasis cases. 6% active lesion + 40% scars | Clinical | L aethiopica | single, ulcerative | 0.63:1 (population) |
| Milosev, 1969 | Sudan | Case series | ENT department | 12/16 from an endemic zone (kala-azar) | 16 | NA | Smear + Animal inoculation | NA |  | Only male |
| Morrone et al, 2011 | Ethiopia | Case series | Italian Dermatological Centre | Dermatology patients | 471 | 15HIV/186LC | Smears + biopsy | NA | 86%LCL,11%mucosal, | 2.5:1 |
| Ndiaye et al, 1984 | Senegal | Case series | Dermatology dakar and polyclinic Cap-Vert | Dermatology patients | 260 cases (1973 : 25, 1974 : 27, 1975 : 32, 1976 : 25, 1977 : 42, 1978 : 41, 1979 : 33, 1980 : 35) |  | NNN, vaccinostyle | NA | polymoprh, different between balck and white-there is halo, often multiple and ulcerated, | NA |
| Negera et al, 2008 | Ethiopia | Cross-sectional | Community | Kibet 5291, Woliya 6029, Boze 5723 (population). |  | 92/1907 active cases. | NNN, biopsy, skin scrapings | L aethiopica | 46.7%has single | 0.92:1 (Not SS) |
| Ngouateu et al, 2012 | Cameroon | Cross-sectional | Community |  |  | 146/32466 active cases, 261/32466 had scars. 4.8% with HIV among the 146 | Parasitological | L major | nodule with or without ulcers, multiple | 0.95:1 (infected) |
| Niamba et al, 2006 | Burkina Faso | Descriptive & prospective study | dermatology service of CHU YO | HIV patients | 32 cases | NA | Smear | L major asssumed | classic but atypical, unusual and atypical | 01:01 |
| Obasi et al, 1991 | Nigeria | Case series | Ahmadu Bele University Teaching Hospital | Dermatology patients |  | 21/18000 in dermatology | Clinical + physical diagnosis | NA | papular, fungating, ulcers non healing-dry with satellite papules | 2:1 (infected) |
| Okwori et al, 2001 | Nigeria | Cross-sectional | School | School-children | 10226 | 394/10226 active lesions. 6.8% of active lesions and/or scars (overall prevalence) | On-sight clinical case detection + parasitological examination | NA | dry and moist types | NA |
| Oliveira et al, 2009 | Mali | Cross-sectional | Community | 663 from Kemena, 867 from Sougoula |  | LST + :: Kemena : P 45.4%, I1 18.5%, I2 17.0%; Sougoula P19.9%, I1 5.7%, I2 5.7% | LST | NA |  | 0.89:1 |
| Padovese et al, 2009 | Ethiopia | Case series | Italian Dermatological Center | Clinically suspected patient. Schoolchildren (45%), farmes (33%) |  | 5.6% HIV among the Leish. | Biopsy + parasiitological diagnosis | NA | LCL 109 | 3.05:1 (infected) |
| Pampiglione et al, 1977 | Guinea | Cross-sectional | Community | Near rail and port | 388 | LST 14.7% | Physical examination | NA | NA | Only male |
| Sang et al, 1993 | Kenya | Case series | Referral hospitals + community | Population + patients |  | 53/11167 lesions, 28/11167 scars** | NNN, physical examination | L tropica | large non ulcerous if long duration. | NA |
| Sang et al, 1993 | Kenya | Cross-sectional | Community | Children from a sedentarity farming communities |  | 18 New cases/18528 population : >1900m 0/8725, <1900m 18/9803. | Smears + physical examination + NNN | all 3 | NA | NA |
| Sang et al, 1994 | Kenya | Cross-sectional | Community | temporary settlers, migrant workers | Residents : 425 | 83/167 active lesions, 30/167 scars | Smear + NNN | L aethiopica | NA | 7.33:1 (sample), 11.7:1 (infected) |
| Sarojini et al, 1983 | Ethiopia | Case series | ALERT Addis Ababa | Patients | 104 cases | NA | Smear + biopsy + NNN | L aethiopica assumed | 98 with LCL | 1.6:1 |
| Seid et al, 2014 | Ethiopia | Cross-sectional | Community | NA | NA | NA | Smear + culture | NA | NA | NA |
| Traoré et al, 2001 | Burkina Faso | Case series | Health facilities in Ouagadougou | Patients | 1845 cases (1996 : 61, 1997 : 55é, 1998 : 1218) | NA | Smear | L major in 1 | ulcerocrusted form | 0.99:1 |
| Traore et al, 2016 | Mali | Cross sectional | Community |  | NA | 5.5/1000 active case | NNN, biopsy and smears | NA | NA | 0.5:1 |
| Wilkins et al, 1972 | Ethiopia | Cross sectional | Civil Hospital | Farmers | 21 | 0.22% LST+ | Smear + histopathology | L major assumed | crusted40%; papuloulcerated16% | 9.5:1 |
